# Oral Manifestations in Patients in Treatment with Antidepressants: A Systematic Review

**DOI:** 10.3390/jcm13226945

**Published:** 2024-11-18

**Authors:** Juan Manuel Alcázar-Hernández, Miguel Ramón Pecci-Lloret, Julia Guerrero-Gironés

**Affiliations:** Gerodontologý an Special Care Dentistry Unit, Morales Meseguer Hospital, Faculty of Medicine, University of Murcia, 30008 Murcia, Spain; juanmanuel.alcazarh@um.es (J.M.A.-H.); julia.guerrero@um.es (J.G.-G.)

**Keywords:** antidepressants, oral health, xerostomia, dental caries, periodontal disease, SSRIs, tricyclic antidepressants, taste dysfunction, oral bleeding complications

## Abstract

**Background/Objectives**: The rising use of antidepressants is linked to oral health risks, including xerostomia, caries, and periodontal disease. Recognizing these risks is essential for improving patient care. To systematically review oral manifestations in patients undergoing antidepressant treatment. **Methods**: This review follows the PRISMA guidelines and includes observational studies published in the last 21 years. A PICO-based question was developed to select relevant studies, which were assessed for quality using a modified STROBE checklist. **Results**: A total of 11 studies were analyzed, revealing a consistent association between antidepressant use and the increased risk of xerostomia, caries, and periodontal disease. Additional findings included taste dysfunction and oral bleeding complications. Among the antidepressants, selective serotonin reuptake inhibitors (SSRIs) and tricyclic antidepressants (TCAs) were most commonly associated with xerostomia and caries. However, no significant impact was observed on the chemical composition of saliva or on hemostasis in invasive dental procedures. **Conclusions**: Antidepressant use may lead to significant oral health issues, notably xerostomia and caries. Further studies are recommended to clarify the influence of specific antidepressants and confounding factors, such as treatment duration, dosage, and hygiene habits, on oral health outcomes.

## 1. Introduction

In a society where stress is on the rise and the search for a better quality of life is constant, psychic disorders such as depression are increasingly common, affecting 3.7% of the population [1,2]. This has led to an increase in the use of antidepressants, with one in six people consuming some type of psychodrug [3,4]. Depression, especially among young people, has increased considerably, being twice as prevalent in women [5].

Modern antidepressants may slightly reduce the symptoms of depression compared to placebos, although their effectiveness varies due to the heterogeneous nature of depression [6]. A gradual therapeutic approach is suggested, starting with social and psychological interventions, and then moving to more specific therapies and medicines [7]. Over time, several types of antidepressants have developed. Initially, in the 1950s, monoamine oxidase inhibitors (MAOIs) emerged, followed by tricyclic (TCAs) and tetracyclic antidepressants in the 1980s [8,9]. In 1987, selective serotonin reuptake inhibitors (SSRIs) appeared, such as citalopram and fluoxetine, which are the most commonly used today for their efficacy and safety [2,8].

Antidepressants not only treat depression, but also other disorders such as obsessive–compulsive disorder (OCD), anxiety, eating disorders, and irritable bowel syndrome, among others [2,9]. Although effective in 50–70% of patients, their latency time may lead to the discontinuation of treatment or increased risk of suicide [8]. Each class of antidepressants operates through distinct mechanisms of action, which lead to specific therapeutic effects as well as varied side effect profiles. This diversity in action and side effects underscores the importance of understanding the potential impacts of each type of antidepressant on oral health (Table 1). Antidepressants are mainly metabolized in the liver and may have temporary side effects such as anxiety, insomnia, and sexual dysfunctions, in addition to some that are more serious, but less common [2,9]; for example, SSRIs are associated with the risk of hyponatremia (low sodium level in the blood), which corresponds to the elderly but also the concomitant use of diuretics (Table 1). In summary, antidepressant use should be controlled and monitored due to its potential side effects and variability in patient response.

Recent studies emphasize the impact of antidepressants on oral health, particularly on periodontium [10], caries, and xerostomia [11]. SSRIs, for example, are associated with increased periodontal disease risk due to their effects on bone density and inflammation [10]. Additionally, TCAs and other antidepressants with anticholinergic properties often lead to xerostomia, which reduces salivary flow and increases caries risk [11].

The increase in recent years of patients using antidepressants, and the knowledge that these drugs can cause alterations in the oral cavity evidence the need to perform a systematic review that synthesizes the oral manifestations that can occur in this type of patient. In this way, this article aims to make a qualitative synthesis of the scientific literature to identify the possible oral manifestations that patients treated with antidepressants may suffer.

The general objective of this systematic review is summarized in a qualitative synthesis of the available scientific information, by searching the literature in various databases, in order to identify the possible oral manifestations that could occur in people treated with antidepressants. The specific objectives are as follows: identify the most common oral condition in people treated with antidepressant drugs; indicate which type of antidepressants are most related to oral manifestations; and know which factors can influence the appearance of oral problems in people who use antidepressants.

## 2. Materials and Methods

This systematic review was conducted in accordance with the PRISMA 2020 guidelines (Preferred Reporting Items for Systematic Reviews and Meta-Analyses) and was registered in the PROSPERO database (International Prospective Registry of Systematic Reviews) under the identification number CRD42023482042. The research question was formulated using the PICO framework as follows: What are the oral manifestations observed in patients undergoing treatment with antidepressant drugs? (P: human subjects; I: treatment with antidepressant drugs; C: individuals not receiving antidepressant treatment; and O: prevalence of oral manifestations in those treated with antidepressants). The search strategy, study selection, data extraction, and quality assessment (including the risk of bias evaluation) were carried out independently by two researchers (J.M.A.-H. and J.G.-G.). In cases where discrepancies arose, a third investigator (M.R.P.-L.) was consulted for resolution.

### 2.1. Search Strategy

The search strategy was conducted in December 2023 using five electronic databases: MEDLINE, Web of Science, Scopus, Cochrane Library, and SciELO. The search was restricted to the studies published between January 2003 and December 2023 across all the databases. The terms listed in Table 2 were used to build the strategy, combined with the Boolean operators “OR” and “AND”. Additionally, advanced search tools, such as the truncation symbol (*), were applied to refine the results.

### 2.2. Eligibility Criteria

Regarding the eligibility criteria, which were defined according to the research question and the objectives of the study, we found the following: articles that study oral disorders and include patients treated with antidepressants; conducted on humans; written in English or Spanish; published in the last 20 years; and articles that are observational (cohorts, case–control, descriptive, and longitudinal) and experimental studies.

### 2.3. Study Selection

The references collected through the search strategy were imported to the EndNote appointment manager (Clarivate Analytics, London, UK) to remove duplicates. A selection process was then conducted by initially reviewing the titles and then summaries following the inclusion and exclusion criteria. Eligibility was then assessed and a qualitative synthesis of the articles that met these criteria was carried out through a thorough review of the full text.

### 2.4. Design of the Study

For the bibliometric analysis, the data collected from each article included the author, year of publication, the journal, and the country where the study was conducted. A summary table was also created to organize key details such as the author and publication year, study design, sample or study groups, participants’ ages, types of antidepressants administered, observed oral manifestations, significant findings, and conclusions.

### 2.5. Quality Analysis

An adapted version of the STROBE (Strengthening the Reporting of Observational Studies in Epidemiology) checklist was utilized to assess the risk of bias in the selected studies [12]. This adaptation focused on 11 specific criteria corresponding to items 5, 6, 7, 8, 10, 12, 14, and 15 from the original STROBE checklist. Compliance with each criterion was marked with a check (✓), while non-compliance was indicated with a cross (✗). Based on the total score, the studies were categorized as follows: a low risk of bias for scores between 8 and 11 points, a moderate risk for scores between 4 and 7 points, and a high risk for scores of 3 points or fewer.

## 3. Results

### 3.1. Selection of Studies and Flowchart

**Figure 1:**
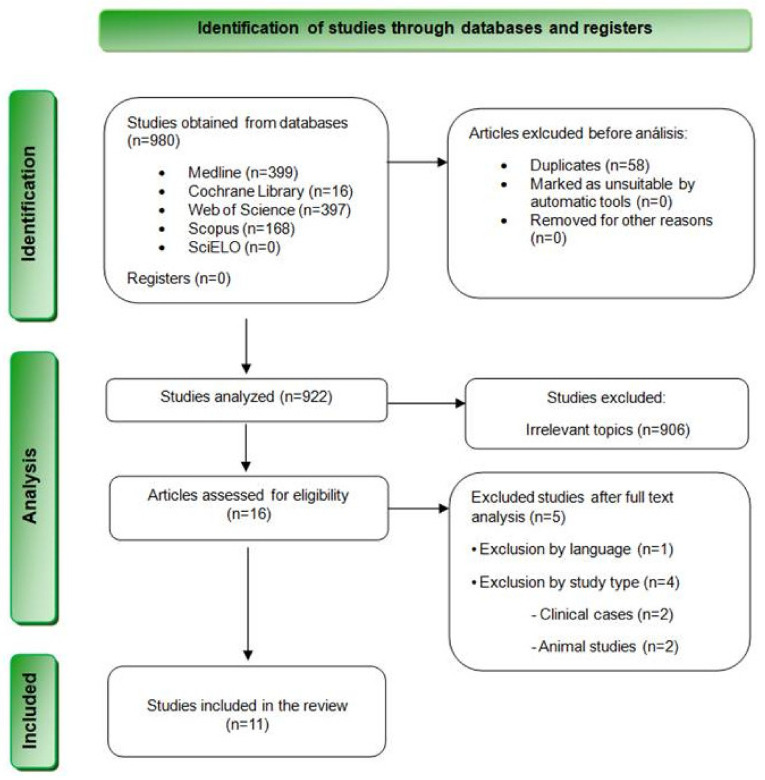
Flowchart depicting the inclusion of studies in this systematic review based on the PRISMA 2020 Statement.

The bibliographic search resulted in a total of 980 results. Specifically, 399 articles were found in MEDLINE (PubMed), 397 in Web of Science, 168 in Scopus, 16 in Cochrane Library, and none in SciELO. Table 3 summarizes the results obtained from each database. Subsequently, 58 duplicate articles were removed, and 922 articles were selected for the revision of titles and summaries. At this stage, 853 articles were excluded after reviewing their titles, and 53 additional articles were discarded after reading their summaries and verifying that they did not meet the inclusion criteria. Finally, the remaining 16 articles were evaluated through a complete reading of their texts. Finally, 5 studies were eliminated after reading them in full text, and 11 articles were finally chosen for qualitative analysis (Figure 1).

### 3.2. Quality Analysis

The quality assessment method used in this systematic review is based on an adapted version of the STROBE guide [12] for observational studies as shown in Table 4 The results of the analysis indicate that eight [13,14,15,16,17,18,19,20] of the works were considered low risk of bias, representing 72.72% of the total. Two studies were rated as moderate risk [21,22], which constitutes 18.18%, and another of the studies was identified with a high risk of bias [23], 9.09%, and was discarded in the extraction of data from the results and discussion of this work (Table 4).

### 3.3. Characteristics of the Studies

#### 3.3.1. Bibliometric Analysis

**Figure 2:**
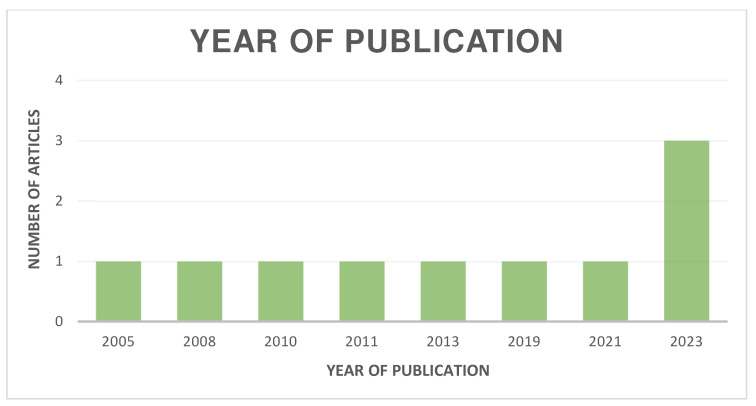
Organization of articles according to their year of publication.

**Figure 3:**
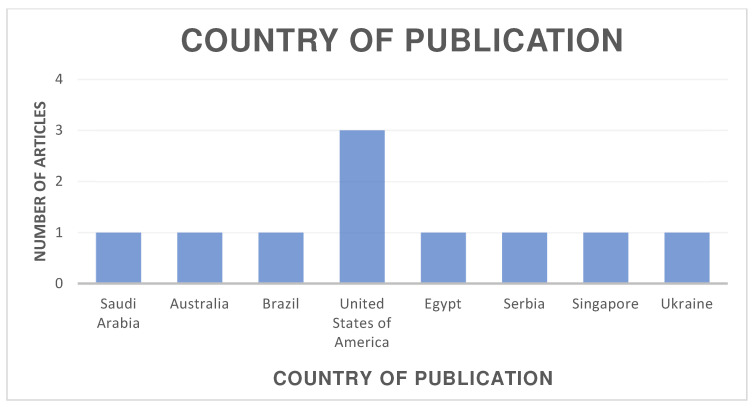
Organization of articles according to their country of publication.

**Figure 4:**
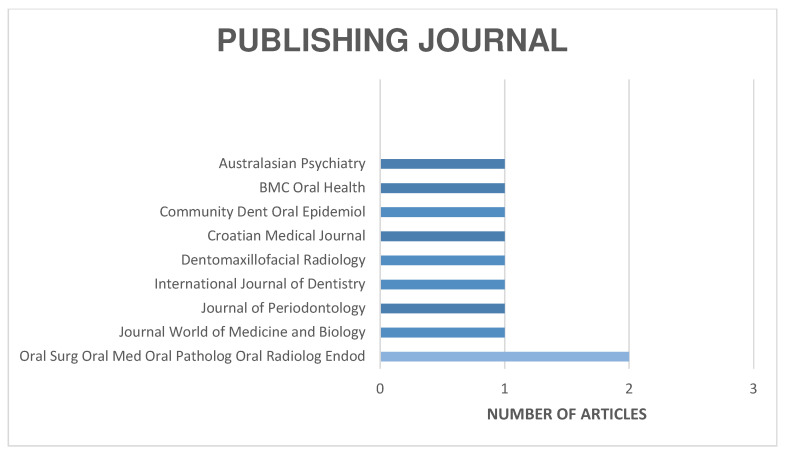
Organization of articles according to their publication journal.

The distribution of the selected articles by publication year is shown in Figure 2, by country in Figure 3, and by journal in Figure 4.

#### 3.3.2. Design of the Study

Within the studies selected for review, the following study designs were identified: five case–control studies [13,14,15,17,19], 50% of the 10 articles selected; three cohort studies [18,19,21], representing 30% of the total; and two cross-sectional studies [16,22] representing 20% of the total (Table 5).

#### 3.3.3. Groups or Sample

The sample size was very variable among all the articles, with five studies that exceeded 100 people sample [13,15,17,19,22], and five articles [14,16,18,20,21,24] that did not exceed that sample amount (Table 5).

#### 3.3.4. Type of Antidepressant

As for the type of antidepressant, we can observe that two studies [15,16] do not specify the specific type of antidepressant patients receive from the study, representing 20% of the articles. As part of the remaining 80%, we found eight articles in which serotonin reuptake inhibitors (SSRIs) are studied, [13,14,17,18,19,20,21,22]. In the same way, we can see that in three articles [13,18,22] of these eight, tricyclic antidepressants (TCAs) are studied together with SSRIs, accounting for 30% of the total. Finally, in just one article [13], which represents 10% of the total, were also included patients treated with tetracyclic antidepressants or monoamine oxidase inhibitors (MAOIs) (Table 5).

#### 3.3.5. Age of Participants

As for the age of the participants, in four articles [17,19,20,21], the average age of the studied groups was indicated, accounting for 40% of the total articles, in contrast to two articles [18,22] where it was indicated by an age range, assuming 20% of the total, and finally in the remaining 4 articles [13,14,15,16] it was indicated by an age range and the average age of the participants, accounting for 40% of the total articles included in this review (Table 5).

#### 3.3.6. Oral Manifestations

As we see in Table 5, we found four articles that study the presence of restorations or cavities [13,15,16,22]. Also noteworthy are two articles that study xerostomia in patients treated with antidepressants [14,22]. On the other hand, we found three articles that study periodontal disease (EPO) [15,16,22]. On the other hand, we found two studies that study other oral manifestations such as periodontal and peri-implant status [19,20]; two other articles dealing with the composition of saliva and its restorative capacity [14,20]; an article on oral bleeding complications after invasive dental procedures [21]; another on mandibular bone mineral density [17]; another on other oral syndromes such as burning mouth, sialorrea, or geographic tongue; and finally, an article on gustatory dysfunction [18].

## 4. Discussion

The relationship between mental disorders and poor oral health is a frequent problem in clinical practice and medical research. This review explores how antidepressants of different types affect oral health, particularly in xerostomia, which is the perception of dry mouth and may be associated with a reduction in salivary flow, called hyposalivation [24]. Between 1% and 29% of the world’s population suffer from xerostomia, which affects the ability to speak and swallow, and can lead to caries [25,26].

Several studies analyzed in this review focus on xerostomia. On the other hand, De Almeida’s et al. study (2008) examined 33 patients divided into groups with and without psychotropic medication, finding a significant reduction in salivation in those treated with psychotropic drugs in this study [14]. However, no decrease in saliva was observed in patients treated with SSRIs at recommended doses compared with the control group. In groups treated with antidepressants, 15 cases of xerostomia were detected. This study claims that these drugs do not have as harmful an effect on salivation compared to the data in the literature, but [27] asserts that these medications cause xerostomia by reducing salivary flow. Similarly, other investigators [28] explain, through the neurotransmitter system that regulates salivary flow, how antidepressants decrease it due to their anticholinergic effect.

Another study, [22], surveyed doctors, dentists, pharmacists, and patients treated with antidepressants. A total of 83.9% of the patients reported xerostomia or caries, and a greater decrease in salivary flow was observed in patients treated with CTA (58%) compared with those treated with SSRIs (32%). More than 80% of the patients taking antidepressants had significant oral pathologies, recommending future research that correlates this disease with other variables such as changes in eating behavior, drug doses, and different antidepressant groups.

Periodontal disease, associated with bacteria such as *Porphyromonas gingivalis* [29], varies from gingivitis to periodontitis. Jovanovic and collaborators found that psychiatric patients taking antidepressants showed a higher CPOD index (decayed, missing, and blocked teeth) and plaque (IP) in their article [15]. Lalloo and collaborators observed an average CPOD of 17.7 in medicated psychiatric patients, higher than the Australian state average, with a high incidence of gingival inflammation and untreated caries, as they can demonstrate in their study [16]. Rindal and collaborators [13] concluded that patients taking antidepressants or non-xerogenic medication were more likely to need dental restorations compared to non-medicated ones, suggesting a relationship between antidepressant medication and poor oral care habits. However, these conclusions are confronted with those of Janket et al. [30], which do not find significant adverse effects of xerogenic medication on the caries index or on CPITN.

Taste dysfunctions, such as the loss of taste, are associated with hyposalivation and can affect the quality of life. Antidepressants with anticholinergic effects may reduce salivary flow, decreasing essential ions and enzymes for taste perception [31]. Additionally, alterations in serotonin pathways by some antidepressants may directly impact taste modulation [32]. Mikhail and collaborators [18] studied these dysfunctions in patients treated with antidepressants, finding that 70% of those treated with TCAs, 20% of those treated with SSRI, and those not medicated had taste problems, especially with sweet taste. Other studies, such as Arbisi and collaborators [33], also found a decreased ability to detect sweet taste in people with seasonal affective disorder.

The studies by Kotsailidi and collaborators [19] and Alharthi and collaborators [20] analyze the effects of antidepressants in patients with dental implants. Kotsailidi [19] found a significant association between SSRI use and marginal bone loss in 105 patients, suggesting an increased risk of peri-implantitis and implant failure in SSRI users. Alharthi and collaborators, however, found no significant differences in bone loss or other parameters in their study of 103 implants, provided adequate oral health is maintained [20]. In the same way, other works like that of Wu et al. [34] report implant failure rates more than double (4.6% non-SRIS users and 10.6% SSRI users), highlighting the need for more accurate and scrupulous implant treatment planning in SSRI users. Other cofactors may also contribute to implant failure in patients undergoing antidepressant therapy. Factors such as poor oral hygiene, smoking, systemic conditions like diabetes, and prolonged treatment duration can interact with the effects of antidepressants, potentially increasing the risk of peri-implantitis and bone loss. These cofactors may amplify inflammation and compromise bone density, further impacting the stability and success of dental implants [35].

In the context of mandibular bone mineral density, Gupta and collaborators [17] observed that patients treated with SSRIs had a higher probability of condylar pathology and lower mandibular bone quality, supporting the idea that SRIs negatively affect bone mineralization, as in other studies such as the one by Coşgunarslan et al. [36] which evidence that jaw bone areas with abundant bony trabeculae are harmed by the use of SSRIs. In addition, the use of SSRIs may lead to calcium and vitamin D depletion, increasing fracture risk in older adults, especially those with severe periodontitis. Calcium and vitamin D supplementation is recommended in these cases to help maintain bone health [37]. The effects of SSRIs on bone mineral density (BMD) may be influenced by treatment duration, especially in elderly patients. Additionally, fluoxetine’s anti-inflammatory and immunomodulatory properties could impact periodontal health and increase caries risk, underscoring the need for further research on its effects on oral and bone health [38].

Napeñas and collaborators [21] investigated oral hemorrhagic complications in patients treated with SSRIs, finding a minimal risk of hemorrhagic complications in invasive dental procedures, and concluding that peri- and postoperative precautions are sufficient to avoid problems. However, other studies such as the one by Weinrieb and collaborators [39] suggest that SSRIs can significantly increase the risk of gastrointestinal bleeding, especially when combined with NSAIDs.

Given the findings of this review, future research should focus on identifying preventive strategies to manage oral health risks in patients undergoing antidepressant therapy. Approaches such as the use of salivary substitutes to alleviate xerostomia and remineralizing agents to counteract enamel demineralization could be beneficial. Additionally, long-term studies with larger sample sizes are needed to investigate the cumulative effects of different classes of antidepressants on oral health, especially among individuals with additional risk factors, such as smoking, diabetes, or poor oral hygiene. Such research would provide valuable insights into tailored oral health protocols for this population, ultimately aiming to improve the quality of life and reduce complications associated with antidepressant treatment.

The systematic review has several significant limitations. One of them is the exclusion of many results that did not meet the inclusion criteria. This significantly reduced the number of studies analyzed. In addition, the lack of sufficient studies with comparable data made objective evaluation and the possibility of meta-analysis difficult. Another notable limitation was the restriction to articles written only in Spanish or English and the selection of studies published in the last 20 years. Although we initially considered shorter time ranges, the search was extended until January 2003 due to the lack of studies that met all the established criteria. Another limitation of this study is the difficulty in attributing symptoms like xerostomia solely to antidepressant use, particularly in patients with chronic comorbid conditions such as cancer, diabetes, or respiratory failure. These conditions often involve polypharmacy and can independently cause or exacerbate oral manifestations, making it difficult to isolate the specific contribution of antidepressants versus the underlying illness or the combined effects of both. This overlap underscores the complexity of assessing the impact of antidepressants on oral health in patients with coexisting health issues.

## 5. Conclusions

Antidepressants can cause xerostomia, cavities, taste dysfunctions, the failure of dental implants, and the deterioration of jawbone quality. These effects are linked to the drugs’ mechanisms of action on neurotransmitter pathways, which can influence oral health outcomes. Antidepressants do not affect the composition and pH of saliva or hemostatic capacity in oral invasive treatments. Xerostomia is the most common oral manifestation, with no significant association found between specific antidepressant types and oral manifestations. Factors such as dosage, eating habits, the duration of treatment, a lack of hygiene, and smoking also influence these outcomes. Proactive measures, including regular dental check-ups and the use of salivary substitutes or remineralization agents, may help mitigate these risks. More studies with consistent methodologies are needed to reach firm conclusions.

## Figures and Tables

**Table 1:** Antidepressant drugs, mechanisms of action, and their adverse effects [2,9].

Group	Mechanism of Action	Common Side Effects
SSRIs	Inhibition of SERT	Nausea, diarrhea, dry mouth, sexual dysfunction, initial anxiety, and hyponatremia
SNRIs	Inhibition of SERT and NAT	Headache, insomnia, nausea, diarrhea, anorexia, sexual dysfunction, and increased blood pressure
NDRIs	Inhibition of NAT and DAT	Dry mouth, constipation, nausea, anorexia, insomnia, headache, anxiety, and increased blood pressure
NRIs	Inhibition of NAT	Anorexia, insomnia, dizziness, anxiety, dry mouth, constipation, nausea, and sexual dysfunction
MTAs	Agonism of MT1/MT2	Nausea, dizziness, drowsiness, and headache
α2 Antagonists	α2 Antagonism	Increased appetite, dry mouth, constipation, sedation, dizziness, and hypotension
SARIs	Inhibition of SERT and 5HT2A/2C Antagonism	Nausea, edema, blurred vision, dry mouth, constipation, dizziness, sedation, and hypotension
TCAs	Inhibition of SERT and NAT	Blurred vision, constipation, increased appetite, dry mouth, nausea, diarrhea, fatigue, sedation, sexual dysfunction, and increased blood pressure
MM	Inhibition of SERT, 5-HT1A/1B/1D Agonist, and 5-HT7 Antagonist	Nausea, headache, dizziness, and dry mouth

SSRIs: selective serotonin reuptake inhibitors; SNRIs: Serotonin–Norepinephrine Reuptake Inhibitors; NDRIs: Norepinephrine–Dopamine Reuptake Inhibitors; NRIs: Norepinephrine Reuptake Inhibitors; MTAs: Melatonergic Agonists; α2 Antagonists: Alpha-2 Antagonists; SARIs: Serotonin Antagonist and Reuptake Inhibitors; TCAs: tricyclic antidepressants; MM: Multimodal Antidepressants; SERT: Serotonin Transporter; NAT: Norepinephrine Transporter; DAT: Dopamine Transporter. MT1/MT2: Melatonin Receptors; α2: Alpha-2 Adrenergic Receptor; 5HT2A/2C: Serotonin Receptors 2A and 2C; 5-HT1A/1B/1D and 5-HT7: Subtypes of Serotonin Receptors.

**Table 2:** Search strategy.

Field 1	(antidepressant* OR “selective serotonin reuptake inhibitors” OR SSRIs OR “serotonin antagonist* and reuptake inhibitor*” OR SARI* OR “noradrenaline-dopamine reuptake inhibitor*” OR NDRI* OR “noradrenaline reuptake inhibitor*” OR NARI* OR “heterocyclic antidepressant*” OR “monoamine oxidase inhibitor*” OR MAOI OR “heterocyclic antidepressant*” OR “serotonin-noradrenaline reuptake inhibitor*” OR SNRI* OR “noradrenergic and specific serotonergic antidepressant*” OR NASSA*)
	AND
Field 2	(“oral manifestation*” OR “oral health” OR “oral disease*” OR “oral patholog*”)

**Table 3:** Results obtained from each database.

Database	Search Strategy	Results
MEDLINE	#1	146.065
	#2	90.873
	#1 AND #2	399
SciELO	#1	4.012
	#2	13
	#1 AND #2	0
Cochrane Library	#1	20.185
	#2	5.470
	#1 AND #2	16
Web of Science	#1	252.741
	#2	58.475
	#1 AND #2	397
Scopus	#1	169.139
	#2	49.077
	#1 AND #2	168

**Table 4:** Results of the quality analysis of the selected studies.

Methods			Rindal y cols. [23]	De Almeida y cols. [24]	Jovanović y cols. [25]	Napeñas y cols. [26]	Lalloo y cols. [27]	Gupta y cols. [28]	Mikhail y cols. [29]	Gandhi y cols. [30]	Kotsailidi y cols. [31]	Alharthi y cols. [32]	Khaitovych y cols. [33]
Configuration	1	Describe the setting, locations, and relevant dates, including periods of recruitment, exposure, monitoring, and data collection.	✗	✗	✓	✗	✓	✗	✓	✓	✓	✓	✓
Participants	2	Indicate the eligibility criteria (inclusion and exclusion) (including matched or control groups if applicable).	✓	✓	✓	✓	✓	✓	✓	✓	✓	✓	✓
	3	Describes the history of antidepressant medication.	✓	✓	✓	✓	✓	✓	✓	✓	✓	✓	✓
Variables	4	Clearly defines the oral manifestation and its diagnostic criteria.	✓	✓	✓	✓	✓	✓	✓	✗	✓	✓	✗
Data sources/measurement	5	Details the methods of evaluation (measurement) of oral expression.	✓	✓	✓	✓	✓	✓	✓	✗	✓	✓	✗
Study size	6	Explain how the study size was arrived at.	✓	✓	✓	✓	✓	✓	✓	✓	✓	✓	✓
Statistical methods	7	Describe all the statistical methods, including those used to control for confounding factors.	✓	✓	✓	✗	✓	✓	✓	✗	✓	✓	✗
	8	Describes any method used to examine subgroups and interactions.	✗	✓	✓	✗	✗	✓	✓	✗	✓	✓	✓
Results													
Descriptive data	9	Provides characteristics of study participants (e.g., demographic, clinical, and social) and reports on exposures and potential confounders.	✓	✓	✓	✓	✓	✓	✓	✓	✓	✓	✓
	10	Please indicate the number of participants with missing data and explain how this was addressed.	✗	✗	✗	✗	✗	✗	✗	✗	✗	✗	✗
Result data	11	Report numbers in each exposure category or summary measures of exposure.	✓	✓	✓	✓	✓	✓	✓	✓	✓	✓	✓
FINAL SCORE AND RISK OF BIAS [High (H), Moderate (M), and Low (L)]			8 (L)	9(L)	10 (L)	7 (M)	9 (L)	9 (L)	10 (L)	6 (H)	10 (L)	10 (L)	7 (M)

**Table 5:** Results of Table 4: results of the articles included in the systematic review.

Author and Year	Study Design	Groups or Samples	Age of Participants	AD Type	Oral Manifestation	Results of Interest	Conclusions
Rindal y cols. 2005 [23]	Cases and controls	n = 7720 ADG = 915 NXMG =5.622 NMG = 1.183	≥55/62 NXMG = 61 NMG= 59	SRIS TCA Tetracyclic MAOI	Dental restorations (indicator of dental caries)	RRAG = 0.78 RRNXMG = 0.67 RRNMG = 0.49	Significant association between taking xerogenic medications (AD) and a higher rate of restorations.
De Almeida y cols. 2008 [24]	Cases and controls	n = 33 GI = 17 GII = 16 GIII = 13 GIV = 8	18–35/32.24	SRIS	Xerostomia and saliva characteristics	SSFR was 33.85% lower in EG compared to CG. No significant differences were found in SSFR between groups III and IV compared to the control group. DM affected 37.50% (group II), 38.46% (group III), and 50% (group IV).	DM was associated with a reduction in TFSE and not with changes in CCS. Psychotropic drug use had no significant impact on Q or pH.
Jovanović y cols. 2010 [25]	Cases and controls	n = 372 GC = 186 GE = 186	20–59/48	Not specified	CMID, POD and PI Index	CMID GE: 24.4 and CMID GC: 16.1 POD higher in EG IP GE: 2.78 IP GC: 1.40	The dental health of EG is inferior to that of healthy people (CG).
Napeñas y cols. 2011 [26]	Cohorts	n = 92	51.2	SRIS	OBC after invasive dental procedures	A total of 167 extractions in 110 visits. In addition, one return visit to the clinic and one phone call were recorded mainly due to oral bleeding problems among all patients.	The rate of OBC after invasive dental procedures is minimal in patients treated with SIRS.
Lalloo y cols. 2013 [27]	Cross-sectional study	n = 50	20–83/41 >50% con <40	Not specified	Edentulism, caries and POD	CMID = 17.7 (IC 95% = 16.9–18.5). - 7 Candida infections. - 8% edentulous and 34% with <21 teeth. - Healthy gums (41%), bleeding (14%), with calculus (35%), and with shallow (8%) and deep (2%) pockets.	The oral condition of this specific group within the community is considerably poorer than that of the general population and requires more intensive attention and treatment.
Gupta y cols. 2019 [28]	Cases and controls	n = 122 GC = 48 GE = 64	EG = 35.2 CG = 31.6	SRIS	Mandibular bone mineral density	EG higher incidence of erosion (OR = 2.926, 95% CI) and severe (OR = 19.86, 95% CI), flatter condyle (*p* < 0.001 for the left side, *p* = 0.009 for the right side), greater height of the mandibular ramus (*p* = 0.001) and a greater mandibular cortical width (*p* = 0.032).	SRIS significantly related to TMJ impairments (including IK, presence of condylar pathology, ramus height, and MCW, with IK being the most influential predictor).
Mikhail y cols. 2021 [29]	Cohorts	n = 30	20–50	SRIS TCA	Gustatory dysfunction	Hypogeusia for sweets was more relevant (*p* < 0.041), being more frequent in TCA users (70%) than in SRIS users and the psychotherapy group (20%).	Significant taste dysfunction is most commonly related to eating disorders, followed by SIRS use.
Kotsailidi y cols. 2023 [31]	Cohorts	n = 105 pacientes152 implantes	55.78	SRIS	Marginal bone loss around DI	The increase in MCW was significantly greater in patients using SSRIs, with a mean difference of 0.37 mm, a *p* value < 0.001, and a 95% CI.	The use of SRIS is associated with increased bone loss around osseointegrated DIs that are in function for an average of 3.8 years.
Alharthi y cols. 2023 [32]	Cases and controls	n = 72 CG = 35 EG = 37	GE= 48.7 ± 5.7 GC = 45.3 ± 5.1	SRIS	Periodontal and peri-implant status and salivary IL-1β levels	TSFR EG: 0.11 ± 0.003 mL/min TSFR CG: 0.11 ± 0.003 y 0.12 ± 0.001 mL/min. IL-1β EG: 57.6 ± 11.6 pg/mL IL-1β CG: 34.6 ± 5.2 pg/mL.	EG and CG with healthy periodontal and peri-implant tissue. No significant differences in IL-1β, as long as they maintain strict oral hygiene.
Khaitovych y cols. 2023 [33]	Cross-sectional study	n = 152 31 PC 30 DR 40 D 51 P	74.2%: 25–44 25.8%: 45–60	SRIS TCA	Caries, POD, xerostomaa and MS.	SB was reported by 33 dentists (82.5%). In total, 28 dentists (70.0%) observed the periodic appearance of ulcers on the oral mucosa, and new caries with a total of 24 (60.0%). In addition, 21 dentists (52.5%) noted bleeding and gingivitis.	In total, >80% of patients treated with AD experience a high incidence of caries and POD in the context of DM.

AD: antidepressants; n: total number of patients; ADG: antidepressant group; NXMG: non-xerogenic medication group; NMG: no-medication group; SRIS: serotonin reuptake inhibitors or serotonergic antidepressants; TCA: tricyclic antidepressants; MAOI: monoamine oxidase inhibitors; RRAG: restoration rate in antidepressant group; RRNXMG: restoration rate in non-xerogenic medication group; RRNMG: restoration rate in no-medication group; SSFR: specific salivary flow rate; CG: control group; EG: study group; DM: dry mouth; CCS: chemical composition of saliva; CMID: caries measurement index in permanent dentition (decayed, missing, and filled teeth); POD: periodontal disease; PI: plaque index; OBC: oral bleeding complications; TMJ: temporomandibular joint; OR: odds ratio; CI: confidence interval; MCW: mandibular cortical width; DI: dental implants; TSFR: total salivary flow rate; IL-1β: interleukin 1-beta; PC: pharmacy customers; DR: doctors; D: dentists; P: pharmacists; MS: mucosal status.

## Data Availability

Not applicable.
